# Mechanisms of *in Vivo* Degradation and Resorption of Calcium Phosphate Based Biomaterials

**DOI:** 10.3390/ma8115430

**Published:** 2015-11-23

**Authors:** Zeeshan Sheikh, Mohamed-Nur Abdallah, Ahmed Abdalla Hanafi, Syed Misbahuddin, Haroon Rashid, Michael Glogauer

**Affiliations:** 1 Faculty of Dentistry, University of Toronto, Toronto, ON M5S 3E2, Canada; 2 Faculty of Dentistry, McGill University, Montreal, QC H3A 1G1, Canada; mohamed.abdallah@mail.mcgill.ca; 3 Faculty of Dentistry, Cairo University, Cairo 11553, Egypt; ahmed.hanafy@dentistry.cu.edu.eg; 4 Faculty of Dentistry, Department of Dental Public Health, University of Toronto, Toronto, ON M5S 3E2, Canada; syed.misbahuddin@mail.utoronto.ca; 5 College of Dentistry, Division of Prosthodontics, Ziauddin University, Karachi 75530, Pakistan; drh.rashid@hotmail.com; 6 Matrix Dynamics Group, Faculty of Dentistry, University of Toronto, Toronto, ON M5S 3E2, Canada; Michael.Glogauer@utoronto.ca

**Keywords:** calcium phosphate, degradation, resorption, implantation, *in vivo*

## Abstract

Calcium phosphate ceramic materials are extensively used for bone replacement and regeneration in orthopedic, dental, and maxillofacial surgical applications. In order for these biomaterials to work effectively it is imperative that they undergo the process of degradation and resorption *in vivo*. This allows for the space to be created for the new bone tissue to form and infiltrate within the implanted graft material. Several factors affect the biodegradation and resorption of calcium phosphate materials after implantation. Various cell types are involved in the degradation process by phagocytic mechanisms (monocytes/macrophages, fibroblasts, osteoblasts) or via an acidic mechanism to reduce the micro-environmental pH which results in demineralization of the cement matrix and resorption via osteoclasts. These cells exert their degradation effects directly or indirectly through the cytokine growth factor secretion and their sensitivity and response to these biomolecules. This article discusses the mechanisms of calcium phosphate material degradation *in vivo*.

## 1. Introduction

Calcium phosphate (CaP) cements are used as bone replacement materials and by composition are classified into (i) apatite cements; (ii) apatite-forming cements; and (iii) dicalcium phosphate dihydrate (brushite) cements [1]. There are a variety of CaP compounds that exist (Table 1) and in the fields of maxillofacial and orthopedic surgery, many CaP materials and compounds have gained clinical acceptance for use in bone repair, regeneration, and augmentation applications [2,3,4]. In dental applications, CaP cements are used for periodontal bone defect filling, immediate implant placement, augmentation of deficient alveolar ridges, maxillofacial reconstruction, sinus lift procedures and coatings for dental implants [4,5,6,7,8,9]. The medical applications include but are not limited to spinal fusion, cochlear implants, fracture and bone defect repair, and coating for orthopedic implant devices [10,11,12].

**Table 1:** List of existing calcium phosphate compounds [1,13,14,15,16,17].

Compound Name	Chemical Formula	Symbol	Mineral	Ca/P Ionic Ratio	Density (g/cm^3^)	Solubility at 25 °C (mg/L)
Monocalcium phosphate monohydrate	Ca(H_2_PO_4_)_2_·H_2_O	MCPM	-	0.5	2.23	~18,000
Monocalcium phosphate anhydrous	Ca(H_2_PO_4_)_2_	MCPA	-	0.5	2.58	~17,000
Dicalcium phosphate dehydrate	CaHPO_4_·2H_2_O	DCPD	Brushite	1.0	2.27	~88
Dicalcium phosphate anhydrous	CaHPO_4_	DCPA	Monetite	1.0	2.92	~48
Octacalcium phosphate	Ca_8_(HPO_4_)_2_(PO_4_)_4_·5H_2_O	OCP	-	1.33	2.61	~8.1
α-Tricalcium phosphate	α-Ca_3_(PO_4_)_2_	α-TCP	-	1.5	2.86	~2.5
β-Tricalcium phosphate	β-Ca_3_(PO_4_)_2_	Β-TCP	-	1.5	3.07	~0.5
Amorphous calcium phosphate	Ca_3_(PO_4_)_2_·nH_2_O *n* = 3–4.5; 15%–20% H,O	ACP	-	1.5	3.01	25.6–32.8
Precipitated hydroxyapatite	Ca_10−*x*_(HPO_4_)*_x_*(PO_4_)_6*−x*_(OH)_2−*x*_	PHA	-	1.33–1.67	3.16	Not available
Calcium-deficient hydroxyapatite	Ca_10−*x*_(HPO_4_)*_x_*(PO_4_)_6*−x*_(OH)_2−*x*_ (0 < *x* < 1)	CDHA	-	1.5–1.67	3.16	~9.4
Hydroxyapatite	Ca_10_(PO_4_)_6_(OH)_2_	HA	Hydroxyapatite	1.67	3.16	~0.3
Oxyapatite	Ca_10_(PO_4_)_6_O	OXA	-	1.67	3.20	Not available
Fluorapatite	Ca_10_(PO_4_)_6_F_2_	FA	-	1.67	3.18	~0.2
Tetracalcium phosphate	Ca_2_(PO_4_)_2_O	TTCP	Hilgenstockite	2.0	3.05	~0.7

**Figure 1:**
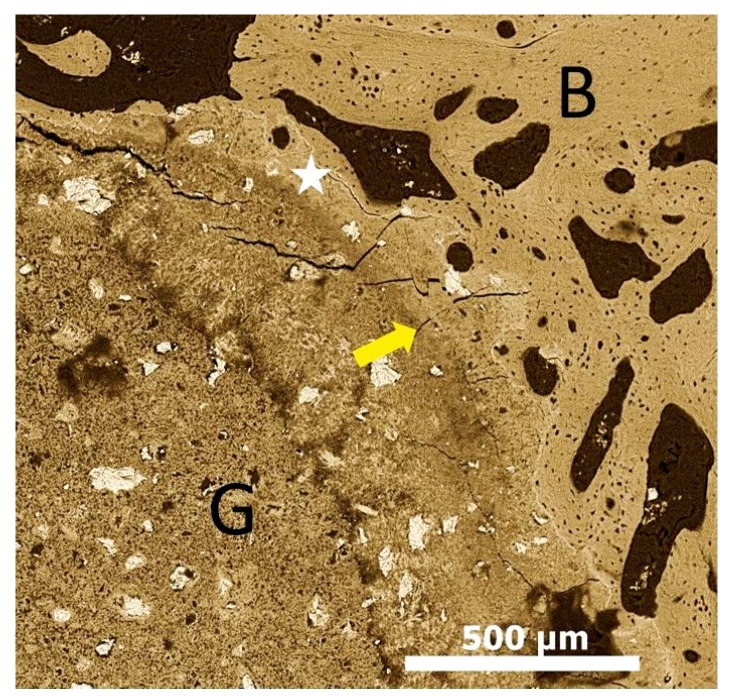
Scanning electron microscope image showing calcium phosphate graft material after 12 weeks osteointegrated with bone and the osteoconduction of bone tissue around the graft material. Graft-Bone interface (Yellow arrow); existing bone (B); graft material (G); Creeping bone substitution/osteoconduction (White star).

For successful bone tissue engineering, it is crucial for the implanted graft materials to have appropriate cellular affinity along with degradation potential. The materials should also have sufficient mechanical strength allowing bone remodeling within a three-dimensional porous structure [18]. The materials should also be fully degradable and this degradation should ideally match with the osteogenic rate [19,20]. A requirement for bone regeneration is the recruitment or presence of osteoblast precursors and growth factors at sites of augmentation. Osteoblast precursors can be provided by the graft material (cancellous autogenous grafts) or by the recipient bed [21]. The early phase of bone regeneration is dominated by active bone resorption and formation throughout the graft. The latter phase of incorporation is characterized by osteoconduction and a process known as creeping substitution (Figure 1) [22,23]. Many of the bone graft materials used today are able to contribute to new bone formation through this biological process [24].

After implantation, biodegradation is critical as this allows for the space to be formed into which the bone and vascular tissues can grow. Biodegradation can be envisioned as an *in vivo* process by which (i) a material breaks down into simpler components, reducing the complexity of chemical compounds by the action of biological systems (cells); (ii) by simple physical breakdown; and/or (iii) chemical erosion [3]. The biological systems can regulate biodegradation via enzymatic or cellular mechanism. The physical breakdown is usually due to passive dissolution of ions and/or disintegration/particulate fragmentation due to loss in mechanical integrity of the implants [2,25]. The chemical alterations in the environment around the implanted materials result in pH level elevation or decrease and can potentially cause erosion. The physical characteristics, chemical composition, crystal structure, and site of implantation play an important role in the biological behavior of CaPs [26,27].

## 2. *In Vivo* Degradation and Resorption of Calcium Phosphates

**Figure 2:**
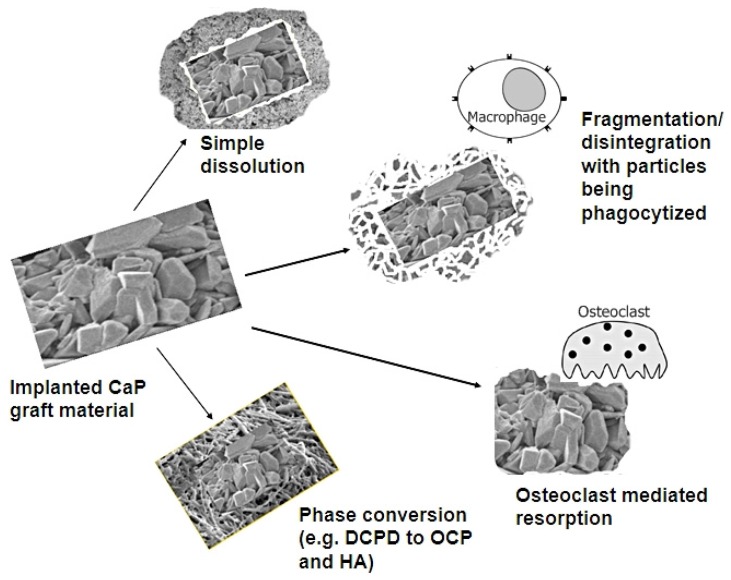
The fate of CaP biomaterials after implantation. (CaP: calcium phosphate; DCPD: Dicalcium phosphate dihydrate; OCP: Octacalcium phosphate; HA: Hydroxyapatite).

For clarity, the term “degradation” represents the physical process of disintegration and fragmentation, whereas, the term “resorption” essentially signifies biodegradation taking place via cellular mechanisms. Biodegradation of CaP based biomaterial is thought to take place via solution-driven extracellular liquid dissolution and cell-mediated resorption processes [28]. The fate of implanted CaP biomaterials is dependent on various mechanisms and processes (Figure 2).

The solubility of the implanted CaP materials heavily affects the dissolution (Table 1) [2,28]. Whereas the disintegration and fragmentation is regulated by the solubility of the necks connecting the particles of cement powder after crystallization [28]. It is believed that the cell mediated CaP resorption (phagocytosis by macrophages) is due to the particle formation as a result of disintegration. Monocytes/macrophages are among the first cells to colonize the biomaterial surface after implantation and play a crucial role in biodegradation [29].

**Figure 3:**
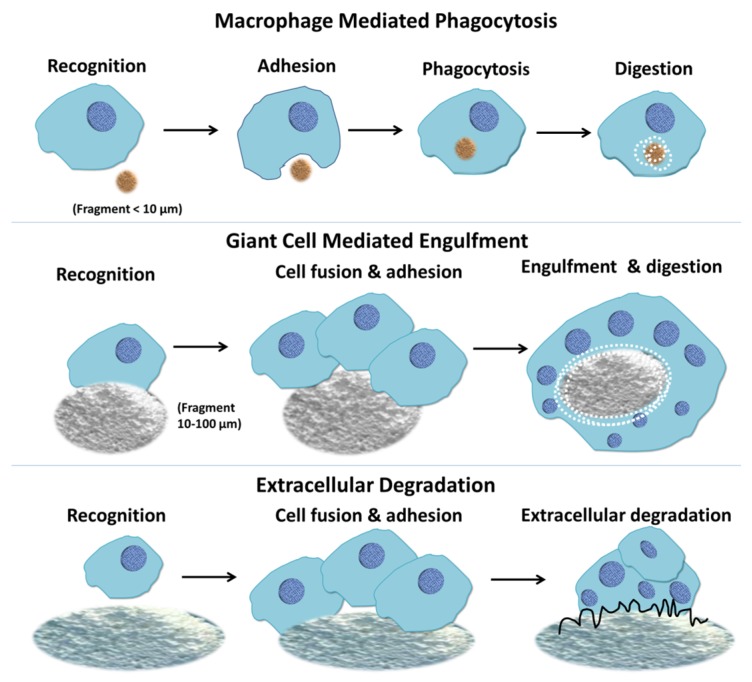
Macrophage response to biomaterials depending on the size of the implanted materials. Macrophages respond to small fragments and particles (<10 μm in diameter) by internalization via phagocytosis and intracellular digestion. If the particle size is larger than 10 μm and smaller than 100 μm, the macrophages fuse together forming giant cells which in turn engulf the particles and digest them. If the particles are larger, the bulk digestion is carried out via extracellular degradation by macrophages and macrophage fused giant cells through release of enzymes and/or pH lowering mechanisms [35].

Biomaterial particles that are generated interact with immune cells (e.g., polymorphonuclear neutrophils and monocytes), leading to cell activation and the release of inflammatory mediators [30,31]. The macrophages or giant cells encounter the CaP particles, attach, and get activated to endocytose [28]. The particle size of the CaP materials implanted affect the rate and effectiveness of cellular resorption activity [32]. The cells that take part in cell-mediated CaP resorption may be osteoclasts, multinucleated giant cells, monocytes, and macrophages directly available in the bone marrow tissue. Phagocytic mechanisms regulated by the monocytes/macrophages or acidic mechanisms via osteoclasts (by reduction of pH in the microenvironment) result in bioresorption of CaP cements *in vivo* [33]. Macrophages respond to small fragments and particles (<10 μm in diameter) by internalization via phagocytosis and intracellular digestion (Figure 3). If the particle size is larger than 10 μm and smaller than 100 μm, the macrophages fuse together forming giant cells which in turn engulf the particles and digest them (Figure 3) [34]. If the particles are larger, the bulk digestion is carried out via extracellular degradation by macrophages and macrophage-fused giant cells through release of enzymes and/or pH lowering mechanisms (Figure 3) [34,35].

Various other cell types such as mesenchymal cells (fibroblasts) present at the implantation site can induce CaP cement solubilization via crystal-cell contacts [33]. Numerous studies have discussed cell mediated resorption of CaPs [28,36,37]. It is seen that for rapidly resorbing cements, it is the macrophages and giant cells that participate actively in the resorption process [38]. In contrast, the slow resorbing cements, osteoclast-type cells are mostly responsible for the cement matrix degradation *in vivo* [37]. Although macrophages loaded with cement particles can be observed throughout the implantation time, they are more prevalent in the resorption zone near the cement border [39].

Multinucleated giant cells have been shown to have a limited capacity to resorb the calcified matrix of the CaP cements [40]. Basle *et al.* have demonstrated that implanted CaP bioceramics induce the recruitment of two multinucleated populations able to degrade the biomaterial implants [41]. The first type associated with the inflammatory reaction (macrophage-polykaryons) intervene immediately after implantation and then disappear. The second type are osteoclasts (corresponding to physiological polykaryons) and are involved in resorption of the calcified cement matrix. The recruitment of this population of cells occurs progressively after implantation [33].

A variety of mesenchymal cells are present at the implantation site of CaP graft materials (e.g., endothelial cells, osteoblasts, fibroblasts, and bone-marrow stromal cells) [33]. If the implanted graft materials are not immobilized to eliminate micro-movements, then these mesenchymal cells result in the fibrous encapsulation of the graft materials [27,33]. This fibrous encapsulation affects the bone formation and biodegradation of CaP materials negatively [42]. Mesenchymal cells are actively involved in the CaP degradation process *in vivo*. It has been shown that the mesenchymal cells can induce the solubilization of CaP scaffolds [33]. Studies have shown that osteoblasts have the capability to phagocytose CaP crystals [43]. Phagosomes containing CaP particles ingested by human bone cells have been observed, and the CaP crystals undergo dissolution within the phagosome [43]. Fibroblasts possess similar ability to internalize CaP particles as shown by osteoblasts [44,45].

It is already known that after implantation, monocytes and macrophages are the first cells to appear during wound healing and are greatly involved in the process of phagocytosis of calcium phosphates. Many growth factors and extracellular matrix proteins are involved in the differentiation and activation of monocyte/macrophage and osteoclast cells [40,46,47,48]. These cells intervene through their cytokine secretions and by their sensitivity to other cytokines [49]. Activity of monocytes can be modulated by many soluble factors and are increased by Interferon gamma (IFN-γ) or 1,25-dihydroxycholecalciferol [50,51], which have been shown to increase their capability to degrade calcium phosphates [48,52]. A study by Laquerriere and co-workers evaluated the inflammatory response to particles with different characteristics (size, shape and sintering temperature) [53]. The most important characteristic appeared to be the shape and the size of the particles, with needle-shaped particles inducing larger production of Tumor necrosis factor-a, Interleukin (IL-6 and IL-10) by cells [53]. Also, the smallest particles induced an increase of the expression and production of the cytokines studied (TNF-a, IL-6 and IL-10) [53]. The crystalline structure and biochemical properties of CaP materials affect the capacity of monocytes/macrophages to produce tumor necrosis factor-α, prostaglandin E2, interleukin 1β, and interleukin-6, which are extensively involved in inflammatory reaction and monocyte and macrophage activation [40]. During early implantation stage, an increase in CaP degradation has been observed the inflammatory reaction intensified by lipopolysaccharides [54,55]. Other molecules such as leukemia inhibitory factor, which is linked with inflammatory reactions and bone remodeling, have shown the ability to reduce the degradation of CaPs [49]. This is believed to take place via the inhibition of phagocytosis, endocytic activity and autophagy. CaP biomaterials once implanted adsorb various proteins (soluble growth factor, serum proteins, and extracellular matrix proteins) onto their surfaces which alter the interfacial properties resulting in enhanced *in vivo* degradation [2,56]. Brushite cements are shown to resorb at a much faster rate when compared to apatite cements [57,58,59]. This difference can be explained by the compositional difference observed for the final products of these cements. Apatite at physiological conditions is the most thermodynamically stable phase and the body fluids are supersaturated with respect to apatite [60]. This supersaturation leads to no dissolution of set apatite cements. Hence, the replacement of apatitic CaP cements with new forming bone tissue can only take place after osteoclast mediated resorption has occurred [61]. Due to the acidic conditions created in the Howship’s lacuna by the osteoclasts, apatite is dissolved similar to bone-remodeling process [61]. Carbonated apatite shows a much higher degradation potential than hydroxyapatite in acidic conditions. Carbonate apatite forms if carbon ions are present during the setting reaction of apatite cement [62]. In contrast to apatite, dicalcium phosphate (DCP) is the most stable phase between the pH of 2.0–4.2 [63]. At physiological pH, brushite is metastable and has the potential to resorb once exposed to body fluid [64]. This means that brushite not only has the ability to be resorbed via osteoclastic activity (long term resorption of brushite cements occurring once the implanted material has undergone phase transformation to apatite), but can also undergo physiochemical dissolution [2,61]. During the first few weeks after implantation brushite appears be resorbed by simple dissolution and more predominantly by cellular activity [39,65,66,67]. The brushite dissolution occurs, leading to the release of loose particles that were initially glued by brushite crystals and these loose particles are then phagocytosed by macrophages. *In vitro* studies have demonstrated the potential for osteoclasts to penetrate brushite cements and demineralize their matrix [68,69]. However, *in vivo* studies have shown that early brushite resorption is regulated by macrophages [68,69,70,71]. Disintegration or fragmentation is a result of dissolution of cements after implantation. It is known that particles released from CaPs can adversely affect the osteoblastic function, viability, proliferation, and extracellular matrix production and can result in peri-implant osteolysis [72]. The smaller the particles are, the stronger the negative effect is seen as the maximum number of particles a single osteoblast can stand is 50 [73].

The presence and inclusion of various ions in the cement during the setting reaction has been shown to have important effects on the reaction and on the final properties of the material in terms of biodegradation and bone formation [2]. An approach towards controlling calcium phosphate cement resorption consists in creating ion-substituted or ion-doped calcium phosphates [74,75], which do not only have a different solubility than the un-doped material, but may provide beneficial bone formation effects due to the release of the doping agents such as strontium (Sr), silicon (Si), magnesium (Mg), potassium (K), carbonate (CO_3_^2−^), and zinc (Zn) during resorption [76]. The incorporation of inorganic compounds in bone replacement materials, which are either constitutional elements of bone or known to influence bone development or regeneration, is an attractive approach [77,78,79,80]. Sr ion is a promising ion that can be delivered by bone substitutes in order to increase bone formation and to decrease bone degradation at the implantation site [81]. Sr-substituted biphasic calcium phosphate material has an effect on the production of cytokines and matrix metalloproteinases (MMPs) by human monocytes [82]. It has already been demonstrated that Sr has a positive effect on bone formation by decreasing MMP-1 and MMP-2 production and increasing type I collagen expression [81]. *In vitro* study has demonstrated anti-inflammatory effects of Sr for human monocytes cultured in contact with calcium phosphates [83]. It has been shown that 1.5% Si-substituted HA enhances the osteoclastic activity *in vivo* [84]. Zn substitution has been found to increase the compressive strength of β-TCP with an inhibiting effect on osteoclast formation or resorption [85]. Incorporation of CO_3_^2−^ in the CaP increases the osteoclast formation by 75% with around 2.5-fold increase in mineral resorption area [86]. Considering the beneficial effects of Sr and Mg, it is believed that their presence in β-TCP will have an influence on osteoclastogenesis and its resorption activity [87]. It has been observed that the addition of certain organic molecules (*i.e.*, citrate ions) and hyaluronic acid slightly decreases the brushite cement resorption rate *in vivo* [88], while cements loaded with collagen tend to re-precipitate into precipitated-hydroxyapatite(Hap) upon incubation in simulated body fluid (SBF) limiting their potential for *in vivo* resorption [89]. Silica gel also has a negative effect on *in vivo* brushite resorption, even though doping β-TCP with Si had no effect [90,91]. The presence of ions via substitution can be used to further research and modify osteoclast function in bone remodeling and thus adjust resorption kinetics of calcium phosphate cements toward bone graft application based on specific application need.

**Figure 4:**
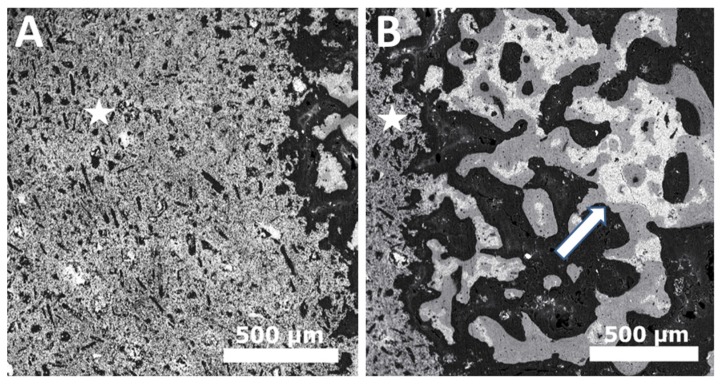
Back scatter scanning electron microscope image of (**A**) Dicalcium phosphate anhydrous (DCPA/monetite) after two weeks of implantation; (**B**) Dicalcium phosphate anhydrous (DCPA/monetite) after eight weeks of implantation showing resorption and replacement of graft material with new bone tissue. [White star indicates DCPA graft material & White arrow indicates remaining graft material (white) being surrounded by new bone (grey)].

A crucial determinant of the solubility and resorption of CaPs *in vivo* is the presence of unreacted phases within the cement matrix. For example, β-tricalcium phosphate (β-TCP) resorbs slower than brushite [92]. Therefore, if brushite cement grafts contain large amounts of unreacted TCP then the dicalcium phosphate dehydrate (DCPD) get resorbed leaving behind long standing β-TCP material [67,92]. Another factor that limits the rate and extent of brushite resorption is the phase conversion phenomenon [57,93]. After demonstrating fast degradation post implantation, the remaining brushite cement converts to less soluble apatites (octacalcium phosphate OCP and hydroxyapatite HA) [94,95,96]. These result in no or very slow resorption from this point onwards mediated solely by osteoclasts, rather than macrophagic phagocytosis [57,65]. Other dicalcium phosphate materials such as monetite show greater resorption and bone formation *in vivo* when compared with brushite cements [97]. The resorption mechanisms for both these chemically similar materials are the same (cellular activity and passive dissolution) [65]. The main reason for this difference in resorption rates is probably due to the fact that monetite cements, unlike brushite, do not undergo phase conversion to apatite and this results in resorption of the cement matrix being replaced by newly forming bone tissue (Figure 4) [2,98].

## 3. Conclusions

An ideal scaffold for bone tissue engineering application should provide initial support for osteoprogenator cells which deposit bone matrix that gets mineralized. For this to happen, the scaffold material should resorb slowly at the same time allowing for the newly forming bone to infiltrate and grow within the scaffold. The degradability and resorption of CaP based biomaterials is not exempt from these requirements if they are to be used with success in clinical situations. The *in vivo* degradation of CaP materials is dependent on the physio-chemical and cellular mechanisms and processes. It can be concluded that a combination of cement dissolution, disintegration, and fragmentation/particle formation followed by phagocytosis through macrophages and osteoclast mediated resorption is responsible for the biodegradation and bioresoprtion of CaPs when implanted *in vivo*. Despite extensive research being conducted, we still do not have a perfect grafting material. Although, CaP have adequate working and setting time, excellent biological properties and the ability to deliver various bone formation enhancing proteins and molecules, they lack adequate mechanical properties and the controlled degradability which is required. Some CaP compounds demonstrate greater biodegradability after implantation than others which can be attributed to the physical characteristics and phase conversion phenomenon to less soluble substrates. The approach required is to develop and use specific CaPs for applications that they are useful for. Further research is required to not only understand the degradation processes of CaP cements better, but also to fine tune the degradation profiles to improve their clinical usefulness and success.
